# Analysis of colonic mucosa-associated microbiota using endoscopically collected lavage

**DOI:** 10.1038/s41598-022-05936-y

**Published:** 2022-02-02

**Authors:** Eiji Miyauchi, Takashi Taida, Masami Kawasumi, Toshifumi Ohkusa, Nobuhiro Sato, Hiroshi Ohno

**Affiliations:** 1 Laboratory for Intestinal Ecosystem, RIKEN Center for Integrative Medical Sciences, Kanagawa, Japan; 2 Department of Gastroenterology, Graduate School of Medicine, Chiba University, Chiba, Japan; 3 Department of Microbiota Research, Juntendo University Graduate School of Medicine, Tokyo, Japan; 4 Immunobiology Laboratory, Graduate School of Medical Life Science, Yokohama City University, Kanagawa, Japan; 5 Laboratory for Immune Regulation, Graduate School of Medicine, Chiba University, Chiba, Chiba Japan

**Keywords:** Microbiome, Gastroenterology

## Abstract

The bacterial composition of the gut lumen and mucosa is distinct and the mucosa-associated bacteria are thought to play a more critical role in interactions with the host immune system. However, limited studies of the gut mucosal microbiota in humans have been available due to methodological challenges. Here, we evaluated the potential use of colonic lavage samples for mucosal microbiota analysis in humans. Among the different types of colonic mucosal samples collected from healthy volunteers, the lavage samples contained a higher amount of bacterial DNA and were less contaminated with host DNA compared to mucosal brushing (brush) and biopsy. Although 16S gene amplicon sequencing showed that the bacterial composition of the lavage was intermediate between that of feces and biopsy, mucosal bacteria abundant in the biopsy were also enriched in lavage samples. Furthermore, differences in mucosal microbes between non-smokers and smokers were detectable in lavage samples. Our data emphasize that colonic lavage is suitable for analysis of the mucosal microbiota. Given its minimal invasiveness and high bacterial DNA content, the colonic lavage will promote research on the human mucosal microbiota, especially in gastrointestinal disorders.

## Introduction

The gut microbiota has been shown to play diverse roles in host homeostasis and diseases. A growing number of studies has demonstrated that patients with diseases, both gastrointestinal and outside the gut, have a distinct gut microbiota composition, or dysbiosis, compared to healthy individuals^1–3^. In addition to disease conditions, genetic background^4^, diet^5,6^ and lifestyle such as exercise^7,8^ and cigarette smoking^9,10^ affect the composition of the gut microbiota. Although the majority of studies on the human gut microbiota have been performed using fecal samples, the gut microbiota is biogeographically different within an individual^11^. Compositionally distinct from luminal microbiota represented by feces^12,13^, the mucosa-associated microbiota interacts more directly with host epithelial and immune cells through pattern recognition receptors and other signals^14,15^. Murine studies have clearly demonstrated that mucosa-associated bacteria but not luminal bacteria induce T helper 17 cells by attaching to epithelial cells in the gut^16–18^, indicating the functional deviation between bacterial communities in the lumen and mucosa. Human studies also have shown the associations between disease conditions or cigarette smoking and altered mucosal microbiota although the mechanism remains to be elucidated^9,19^. Despite the highlighted importance of mucosal bacteria, only limited human studies have focused on the mucosal microbiota rather than fecal microbiota.

The sampling method is a major challenge for analysis of the mucosal microbiota in humans. Small pieces of endoscopic mucosal biopsy collected after bowel preparation with laxatives, such as polyethylene glycerol, has been used as a standard sample for mucosal microbiota. However, the invasive sampling method of a biopsy has a potential risk of unexpected bleeding and infection^20^, which makes it difficult to apply to healthy individuals and patients with extra-intestinal diseases. Alternative sampling methods, such as endoscopic brush sample and colonic lavage, have been proposed for mucosal microbiota analysis^20–22^. As with colonic biopsy, these samples collected after bowel preparation seem to contain enriched mucosal bacteria and the aforementioned risks are reduced with these methods, especially colonic lavage, owing to their minimal invasiveness. Nevertheless, the question remains as to what extent these samples represent the mucosal microbiota, due to the lack of comprehensive comparisons of different sample types of colonic lumen and mucosa. Here, we investigated the quality and quantity of bacterial DNA extracted from feces, colonic mucosal lavage, brush, and biopsy of healthy individuals and also compared the bacterial composition of these samples to evaluate the suitability of lavage for mucosal microbiota analysis. We also conducted a comparative analysis of microbiota between non-smokers and current smokers to ask whether the lavage samples are able to detect compositional changes in the mucosal microbiota between these groups.

## Results

**Figure 1:**
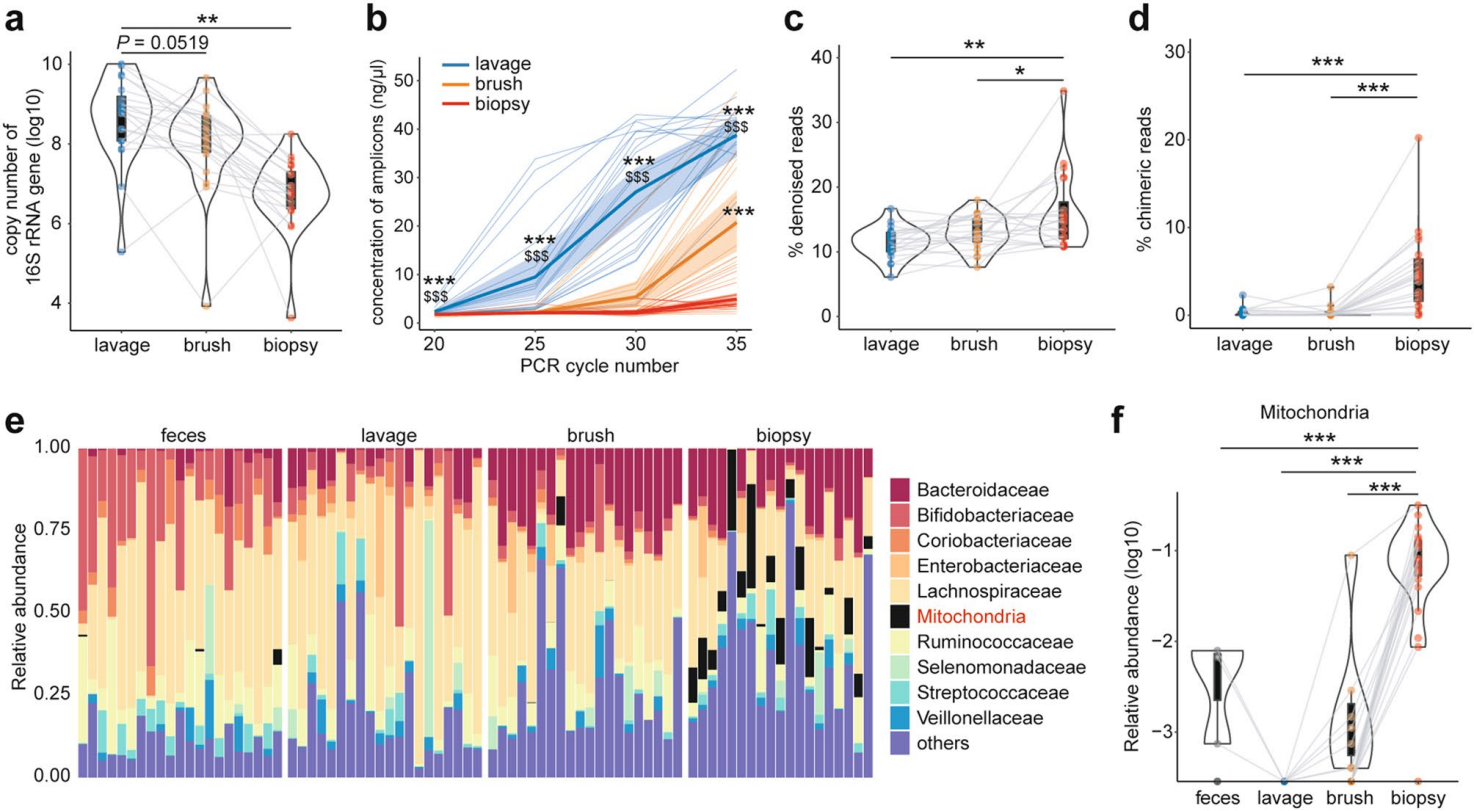
Quality of sequencing data from mucosal samples. (**a**) Bacterial loads in mucosal samples as assessed by qPCR of 16S rRNA genes. (**b**) Concentration of PCR amplicons for 16S rRNA gene sequencing at indicated cycle numbers. The values were calculated per ml for lavage and per sample for brush and biopsy. (**c**, **d**) Percentage of denoised reads as low-quality (**c**) and chimeric reads (**d**), detected and eliminated through the DADA2 pipeline (see Supplementary file: Fig. S1D). (**e**) Barplot representing the top 10 most abundant families and the remainder labeled “others” in feces and mucosal samples. (**f**) Relative abundance of mitochondria. Data represents the mean ± s.d. ***p < 0.001; **p < 0.01; *p < 0.05; one-way ANOVA with Tukey’s test (**a**, **c**, **d**, **f**). ***p < 0.001 vs. biopsy, $$$*p* < 0.001 vs. brush; one-way ANOVA with Tukey’s test (**b**).

We obtained feces and endoscopically-collected colonic lavage, brush, and biopsy samples from 20 healthy subjects for the analysis of microbiota (Supplementary file: Table S1, Fig. S1a). In line with a previous study^23^, the mucus layer, where mucosal bacteria reside, was retained after bowel preparation with a laxative (Supplementary file: Fig. S1b). We first assessed the copy number of the 16S rRNA genes in endoscopically collected samples and found that the lavage samples contained more bacterial DNA than the brush and biopsies (Fig. 1a). Along with this, the efficacy of PCR for constructing sequencing libraries was different among the sample types (Fig. 1b), and a higher number of PCR cycles was required for brush and biopsy samples compared to lavage to prepare enough amplicons for sequencing (Supplementary file: Fig. S1c). It has been reported that higher PCR cycle numbers for library preparation are associated with reduced sequence quality, increased chimera formation and biased microbial composition^24^. Consistent with this scenario, the sequence data obtained from the biopsy contained a higher proportion of low-quality “noise” and chimeric reads compared to data from the other samples (Fig. 1c,d). In addition, reads corresponding to mitochondria were detected in samples other than lavage, especially in the biopsy samples (Fig. 1e,f) after removal of noise and chimeras according to the DADA2 pipeline (Supplementary file: Fig. S1d), probably due to contamination with host mitochondrial DNA that can be amplified by the universal primers for 16S rRNA genes^25^. These results suggest that the low abundance of bacterial DNAs in the biopsy samples leads to increased unwanted reads in the sequence data, while the lavage samples contain enough bacterial DNAs for 16S rRNA gene sequencing and thus overcome this limitation.

**Figure 2:**
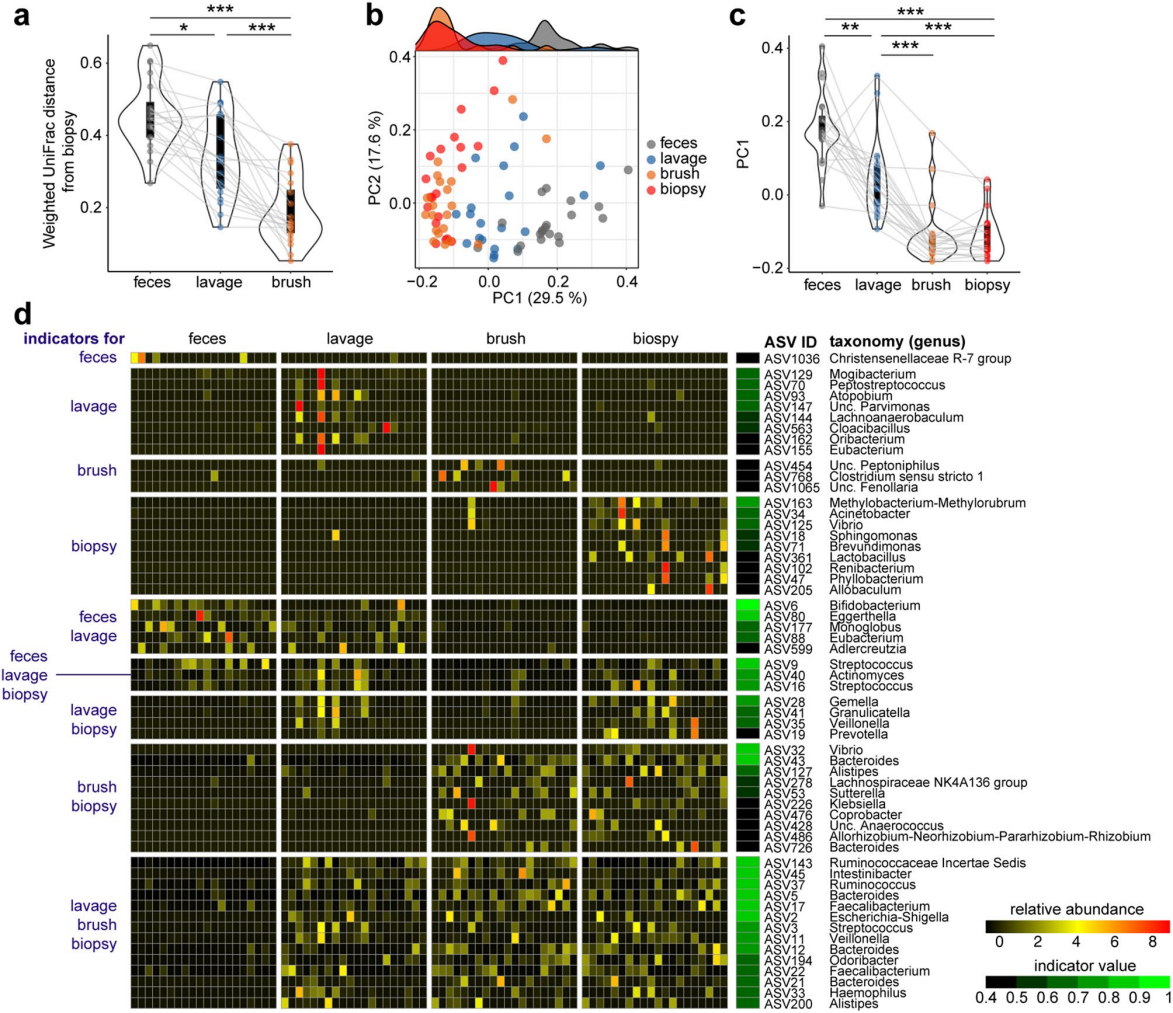
Comparative analysis of bacterial composition in feces and mucosal samples. (**a**) Weighted UniFrac distances from biopsy samples. (**b**, **c**) PCoA plot of weighted UniFrac distances (*R*^2^ = 0.26125, *p* < 0.001, adonis) and the density plot on top shows the sample distribution along the PC1 axis (**b**). The PC1 values of each sample on the plot are summarized in (**c**, **d**) Heatmap of relative abundance and indicator values of indicator ASVs (FDR-adjusted *p* < 0.05, indicator value > 0.4). Data represents the mean ± s.d. ***p < 0.001; **p < 0.01; *p < 0.05; one-way ANOVA with Tukey’s test (**a**, **c**).

We next investigated the bacterial composition of each sample using data from which the reads corresponding to mitochondria and chloroplast (derived from ingested food) DNAs were removed (Supplementary file: Fig. S1d and see “Methods” section). A weighted UniFrac distance from the biopsy samples was much higher for feces compared to lavage and brush (Fig. 2a), indicating that the composition of the mucosa-associated microbiota is distinct from that of the luminal microbiota, as shown in previous studies^12,13^. Principal coordinate analysis (PCoA) based on the weighted UniFrac distances clearly separated the brush and biopsy samples from feces (Fig. 2b), and these samples were clustered and significantly segregated along the PC1 axis (Fig. 2c). The lavage samples showed an intermediate pattern in these data (Fig. 2a–c). The bar plot and biplot of PCoA showed that *Bifibobacteriaceae*, one of the predominant families in the Japanese population^26^, was enriched in feces and contributed to separate the samples along the PC1 axis (Supplementary file: Fig. S2a,b). Furthermore, a ternary plot depicted the individual bacterial families that are differentially abundant in the mucosa (brush and biopsy) and lumen (feces); some of mucosa-enriched families, such as within *Proteobacteria*, were also abundant in lavage samples (Supplementary file: Fig. S2c). Cladograms based on Linear discriminant analysis effect size (LEfSe) provided an overview of distinct taxa in lavage, brush and biopsy samples compared to feces. A similar pattern, except for phylum *Firmicutes* and *Fusobacteriota* (former Fusobacteria), was observed in these sample types (Supplementary file: Fig. S2d).

To uncover more details on the sample type-specific bacteria, we conducted an indicator species analysis at the amplicon sequence variant (ASV) level. In accordance with the biplot (Supplementary file: Fig. S2b), the ASVs annotated as *Bifidobacterium* (belonging to *Bifidobacteriaeae*) and *Coriobacteriaceae* bacteria, such as *Eggerthella* and *Adlercreutzia*, were enriched in feces and lavage samples (Fig. 2d). Of note, the largest cluster consisted of ASVs enriched in the lavage, brush, and biopsy samples with high indicator values (the bottom cluster in Fig. 2d). Taken together, these results suggest that the mucosa-associated bacteria are enriched in the lavage samples.

Due to the low quantity of bacterial DNAs and the contamination of host genomes in the biopsy samples^19,27^, the functional profiles of the mucosal microbiota remain to be determined. We therefore performed an inferred metagenomic analysis using PICRUSt2 to predict whether a bacterial community in the lavage samples displays different functions from that in feces. We observed a considerable number of distinct KEGG orthologues (KOs) and pathways between feces and mucosal samples such as brush and biopsy (Supplementary file: Fig. S3a). These differentially abundant KOs and pathways were also partially detected in the comparison between feces and lavage, although over 30% and 40% of KOs and pathways were distinct in biopsy from lavage and brush, respectively (Supplementary file: Fig. S3b). These results are consistent with a Venn diagram with shared and unique ASVs in which biopsy-specific ASVs overwhelmed the other fractions (Supplementary file: Fig. S3c). These results imply distinct functions between luminal and mucosal microbiota and suggest that the lavage samples, at least partially, could detect functional characteristics of the mucosal microbiota in future metagenomic analysis.

**Figure 3:**
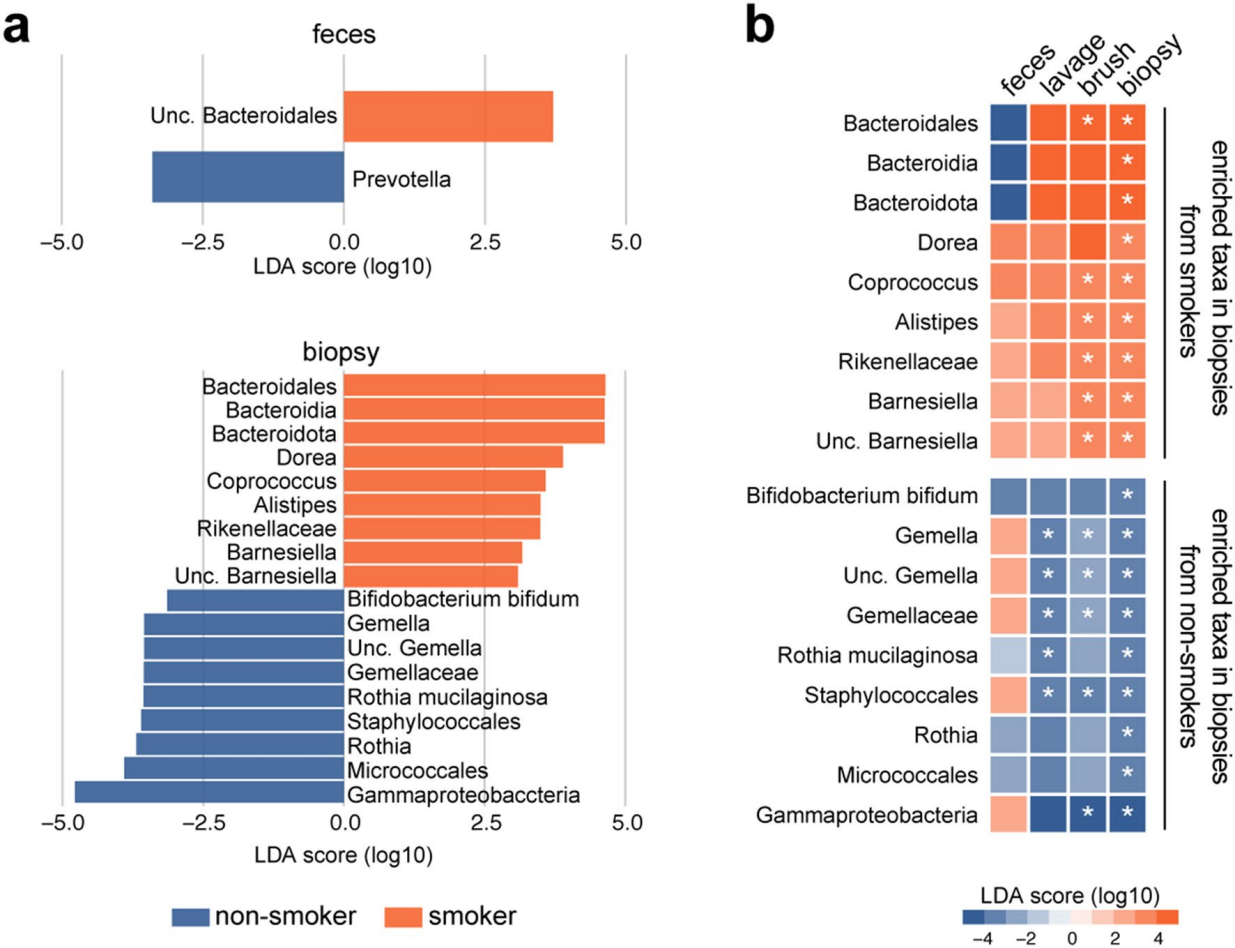
Differences in luminal and mucosal microbiota between non-smokers and smokers. Linear discriminant analysis effect size (LEfSe) analysis of microbiota between non-smokers and smokers. (**a**) Differentially abundant taxa (non-adjusted *P* < 0.05, absolute log 10 LDA score > 3) in feces and biopsy. (**b**) Heatmap of log 10 LDA scores of taxa that are detected in the biopsy samples as discriminants. *p < 0.05; Kruskal–Wallis rank sum test.

We next asked if the lavage is sensitive enough to distinguish the difference in mucosa-associated microbiome composition. To this end we took advantage of the differences in gut microbiota between non-smokers and smokers ^9,10^. LEfSe analysis detected few or no bacterial taxa differentially abundant in the feces between non-smokers and smokers, while 18 taxa were identified in biopsy samples as discriminants between the two groups (Fig. 3a, Supplementary file: Fig. S4). The lavage and brush samples also showed differentially abundant taxa between the groups (Supplementary file: Fig. S4a). The heatmap shown in Fig. 3b summarizes the overlaps of 18 taxa detected in biopsy samples among each sample type and demonstrated that the lavage samples showed an identical pattern of LDA scores to biopsy samples, although they were only partially significant. The coefficient of variation for the relative abundance of smoker-enriched taxa in the lavage samples was higher than those in brush and biopsy (Supplementary file: Fig. S4b), indicating that large variations in bacterial abundance in the lavage may, at least in part, contribute to the increased *P*-values in the comparative analysis.

## Discussion

Although studying mucosal microbiota reveals disease-associated bacteria that are not detectable in fecal samples^19^, the mucosal microbiota has not been well-investigated compared to its fecal counterpart in humans. The need to overcome the challenges and limitations of biopsy sampling to promote research on the mucosal microbiota has been highlighted. Mucosal brushing is less invasive and is also used to investigate the mucosal microbiota; however, this method still carries risk and requires expertise and equipment^20^. Given the minimal^21^ risk and simple sampling procedures, colonic lavage could be the best surrogate for colonic biopsy. We here demonstrated that colonic lavage contains a high yield of bacterial DNAs with enriched mucosal bacteria, indicating the suitability of this sampling method for the analysis of the mucosal microbiota. Given that the lavage samples are collected during colonoscopy without touching the mucosa, as in the case with general medical checkups, this method would be optimal not only for patients with gastrointestinal diseases but also for patients with non-GI diseases and for healthy individuals.

To extend a previous study^21^, we clarified the characteristics of lavage microbiota by comparing them with feces, brush and biopsy. Although a cluster of mucosal bacteria predominant in the brush and biopsy samples were enriched in the lavage, luminal bacteria represented by *Bifidobacteriaceae* and *Coriobacteriaceae* were also abundant in this sample type. This might imply contamination by retained luminal contents in the lavage after bowel preparation and that lavage sampling has a higher contamination risk than the other mucosa sampling methods due to its non-targeted sampling procedure. Further optimization of bowel preparation, such as cleansing agents and timing^28,29^, may minimize the cross-contamination. Unlike previous studies^30,31^, we observed only few or no bacterial taxa that are differentially abundant between non-smokers and smokers in feces, possibly due in part to the small sample size in our study. On the other hand, analysis of mucosal microbiota using the brush and biopsy samples detected a considerable number of discriminants between the groups, implying that cigarette smoking affects the microbiota in the colonic mucosa more that in the other sampled sites, as also demonstrated in the small intestinal mucosa^9^. The lavage samples displayed a similar tendency in the distribution of discriminants, although the statistical significance was observed in limited taxa. This discrepancy among the mucosal samples may be partially explained by a higher coefficient of variation in the lavage samples, which might be caused by the cross-contamination mentioned above. Further studies with a larger sample size and detailed information of subjects (e.g. food-frequency questionnaire or medication history) will provide a deeper insight into the effects of smoking on the mucosal microbiota, which is out of scope from our present study.

It still remains challenging to perform functional analysis of mucosal microbiota by shotgun metagenome sequencing due to limited bacterial density and the contamination of the biopsy with the host genome. Indeed, a recent study found that 90–97% of reads obtained from shotgun sequencing of human intestinal biopsies were mapped onto the human genome^19,27^. Our data revealed that the lavage contains a much lower abundance of mitochondria compared to the other types of samples. In addition, in terms of the quantity of bacterial DNAs, lavage would be more appropriate for shotgun sequencing. We obtained 4–5 ml of colonic lavage from each subject, and DNA extracted from 475 µl of the sample was enough for preparation of 16S rRNA gene amplicons. By increasing the input volume of lavage for DNA extraction and/or by concentrating the extracted DNA, it should be possible to perform the metagenomic analysis. As shown in the inferred metagenome data performed in this studies, the mucosal microbiota might be functionally quite different from the luminal microbiota. Furthermore, the lavage samples also detected the KOs and pathways differentially abundant in the mucosa. Thus, future functional analysis using lavage samples with shotgun metagenome sequencing will lead to a deeper understanding of mucosal microbiota.

## Conclusion

Collectively, our results demonstrate that colonic lavage samples are suitable for analysis of the mucosal microbiota. Given that the lavage sampling procedures carry minimal risk of tissue damage, this method can be readily applied, especially for healthy individuals. In addition, the lavage samples contained a larger amount of bacterial DNAs than the other mucosal samples, which not only contributes to high quality PCR amplicons for 16S rRNA gene sequencing but also shows the potential to be applicable for metagenomic analysis of the mucosal microbiota. Although further optimization of bowel preparation would be needed to prevent cross-contamination with luminal bacteria, the advantages of this method will facilitate the detailed analysis of the mucosal microbiota and deepen the understanding of host-microbe interactions at the mucosal surface.

## Methods

### Study populations and samplings

Healthy adult volunteers who currently smoke cigarettes (smokers) and have never smoked in their life (non-smokers) were recruited from RIKEN and Oriental Ueno Kenshin Center (Supplementary file: Table S1). Participants who had adenomatous polyps were excluded and no abnormality was found with colonoscopy in any of the subjects in this study. Three types of mucosal samples, lavage, brush and biopsy, were colonoscopically obtained from the sigmoid colon after bowel preparation with polyethylene glycol (PEG) according to a standard protocol. The lavage samples (4-5 ml) were collected using a suction trap, avoiding contamination with solid feces. The brush samples were obtained by gentle brushing of mucosal surfaces using an endoscopic cytology brush (Olympus Medical Systems Co. Ltd., Tokyo, Japan) and suspended in 1 ml sterile saline. Two pieces of biopsy (for sequencing and histological analysis, approximately 5 × 5 mm each) were taken using Radial Jaw™ 3 biopsy forceps (Boston Scientific, Tokyo, Japan). Feces were collected at the facilities described above. The samples collected at Oriental Ueno Kenshin Center were transported on dry ice to RIKEN and stored at – 80 °C until analysis. All methods were carried out in accordance with relevant guidelines and regulations. The protocol was approved by the RIKEN Yokohama Campus Ethics Committee (H27-14(4)) and Oriental Ueno Kenshin Center’s Ethics Committee (2019-A1118), and written informed consent was obtained from all participants.

### Alcian blue biopsy staining

One piece of biopsy fixed in Carnoy’s fixative was embedded in paraffin and sectioned at 5-μm thickness. The sections were stained with Alcian blue solution (FUJIFILM Wako Pure Chemical Corporation, Osaka, Japan) for 30 min followed by counterstain with nuclear fast red for 2 min (Vector Laboratories Inc., Burlingame, CA, USA).

### DNA preparation

The overview of DNA preparation is summarized in Supplementary file: Figure S1A. Fecal samples (0.1–0.2 g) were resuspended in methanol, filtered on 100-μm cell strainer, and then centrifuged at 10,000* g* for 10 min. The bacterial pellets (the supernatants were used for metabolomic analysis, data not shown) were air-dried and responded in 475 μl of TE10 buffer (10 mM Tris–HCl, 10 mM EDTA, pH 8.0). The second piece of biopsy suspended in RNAlater was washed with PBS three times and resuspended in 475 μl of TE10 buffer. The colonic lavage, and brush suspension were directly processed with enzymes according to a previous study^32^. Briefly, the suspensions were incubated with lysozyme (15 mg/ml, FUJIFILM Wako Pure Chemical Corporation, Osaka, Japan) and achromopeptidase (2,000 units/ml, FUJIFILM Wako Pure Chemical Corporation, Osaka, Japan). Subsequently, the bacterial cells were further digested with 1% sodium dodecyl sulfate and proteinase K (1 mg/ml, Merck & Co. Inc., USA). The DNA was extracted and purified with phenol:chloroform:isoamyl alcohol (25:24:1) and RNase A (0.1 mg/ml, Nippon Gene, Tokyo, Japan).The degraded DNA was removed using PEG 8000 precipitation. This precipitation step was skipped for the brush and biopsy samples due to the low concentration of DNA and to avoid sample loss. After washing with 70% ethanol, the purified DNA was resuspended in 50 μl of TE buffer (10 mM Tris–HCl, 1 mM EDTA, pH 8.0).

### qPCR analysis

The 16S rRNA gene copy number was quantified by qPCR using SYBR Premix Ex Taq (Takara Bio Inc., Shiga, Japan) and universal bacterial primers 340F (ACTCCTACGGGAGGCAGCAGT) and 514R (ATTACCGCGGCTGCTGGC).

### 16S rRNA gene analysis

The V4 variable region of the 16S rRNA gene was amplified by PCR and the dual-indexed libraries were prepared as described previously^32^. The PCR cycle number was increased from 25 to 35 when the PCR products were not visible on a gel. The double-stranded amplicons were quantified using the Quant-iT PicoGreen ds DNA Assay Kit (Thermo Fisher Scientific K.K., Tokyo, Japan). Sequencing was performed on a MiSeq platform using 250-bp paired-end V2 chemistry (Illumina, San Diego, CA, USA).

Raw data generated from the MiSeq was demultiplexed using bcl2fastq (v1.8.4, Illumina). The resulting fastq files were processed with DADA2^33^ (v1.18.0) using parameters described in the tutorial pipeline (https://benjjneb.github.io/dada2/tutorial_1_8.html). After trimming low-quality reads, the dereplicated reads were used for amplicon sequence variants (ASVs) inference. Chimera-free ASVs were assigned against the SILVA database v138^34^ with a naive Bayesian classifier implemented in DADA2.

Downstream analysis was performed with the phyloseq package (v1.34.0) in R (v4.0.3). After removal of non-targets (mitochondria and chloroplast), relative abundance data were used for calculation of weighted UniFrac distances and principal coordinate analysis (PCoA). Permutational multivariate analysis of variance on weighted UniFrac distances was performed using the adonis function in the vagan package (v2.5.7) with 1,000 permutations. The association between individual factors (age, gender and smoking pack-year) and PCoA ordinations was analyzed using the envfit function in the vegan package with 1,000 permutations. Differentially abundant taxa and ASVs were determined with the indicspecies package (v1.7.9) and linear discriminant analysis effect size (LEfSe)^35^. Inferred metagenomic analysis was performed with PICRUSt2 (v2.3.0)^36^. The differentially abundant KEGG orthologues and pathways were evaluated with the ALDEx2 package (v1.22.0)^37^ using default parameters. All data except for the LEfSe cladogram were visualized by ggplot2 (v3.3.3)^38^, pheatmap (v1.0.12)^39^, ggtern (v3.3.0)^40^, and ggVennDiagram (v0.3)^41^ packages.

### Other statistical analyses

All statistical analyses were performed in R. Normal distribution of data was evaluated with D’Agostino-Pearson’s normality test and differences between groups were tested using one-way ANOVA followed by Tukey’s multiple comparison test. *p* values < 0.05 were considered significant (*p < 0.05, **p < 0.01, and ***p < 0.001).

### Ethics approval and consent to participate

The protocol was approved by the RIKEN Yokohama Campus Ethics Committee and Oriental Ueno Kenshin Center’s Ethics Committee. Written informed consent was obtained from all participants.

## Supplementary Information

Supplementary Information 1.

Supplementary Information 2.

Supplementary Information 3.

Supplementary Information 4.

Supplementary Information 5.

## Data Availability

Raw sequencing data of 16S rRNA gene are available from the DDBJ Sequence Read Archive under DDBJ BioProject identifier PRJDB11319.
