# Supplementary figures and images for: Analysis of colonic mucosa-associated microbiota using endoscopically collected lavage
Source: Sci Rep. 2022 Feb 2;12:1758. doi: 10.1038/s41598-022-05936-y (PMC8810796; doi:10.1038/s41598-022-05936-y)

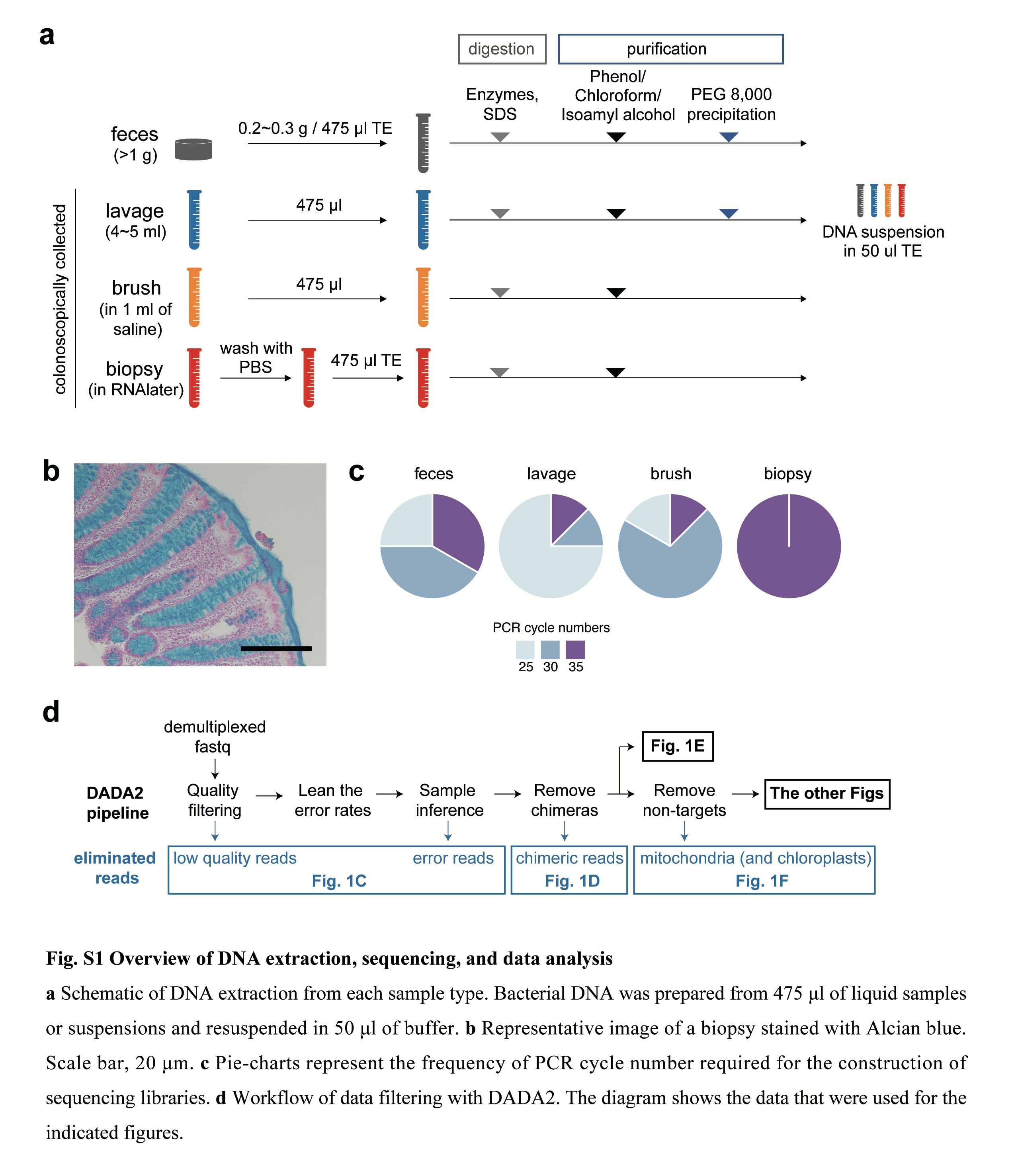

Supplement: Supplementary file 1 — Supplementary Information 1. [file 41598_2022_5936_MOESM1_ESM.tif]

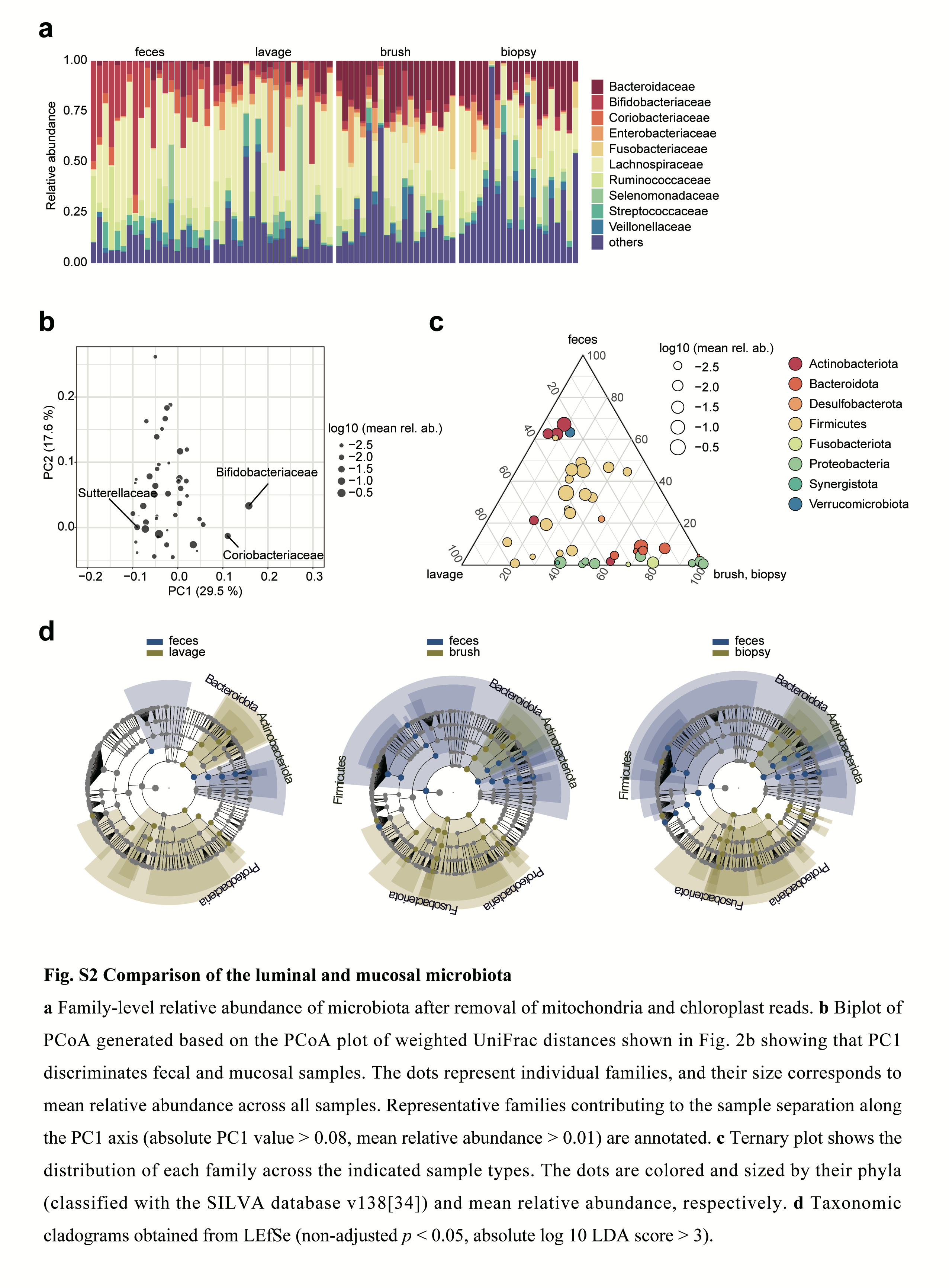

Supplement: Supplementary file 2 — Supplementary Information 2. [file 41598_2022_5936_MOESM2_ESM.tif]

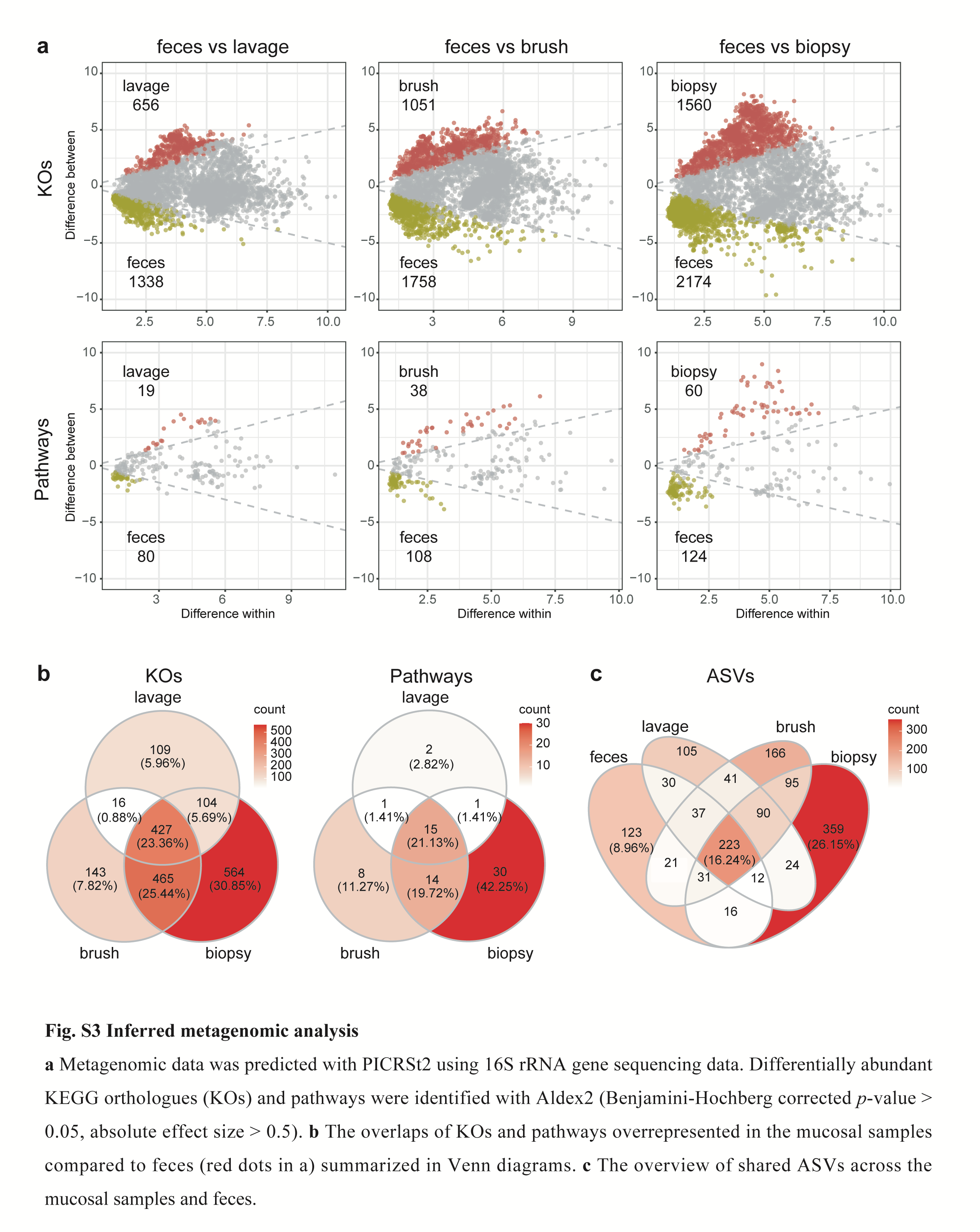

Supplement: Supplementary file 3 — Supplementary Information 3. [file 41598_2022_5936_MOESM3_ESM.tif]

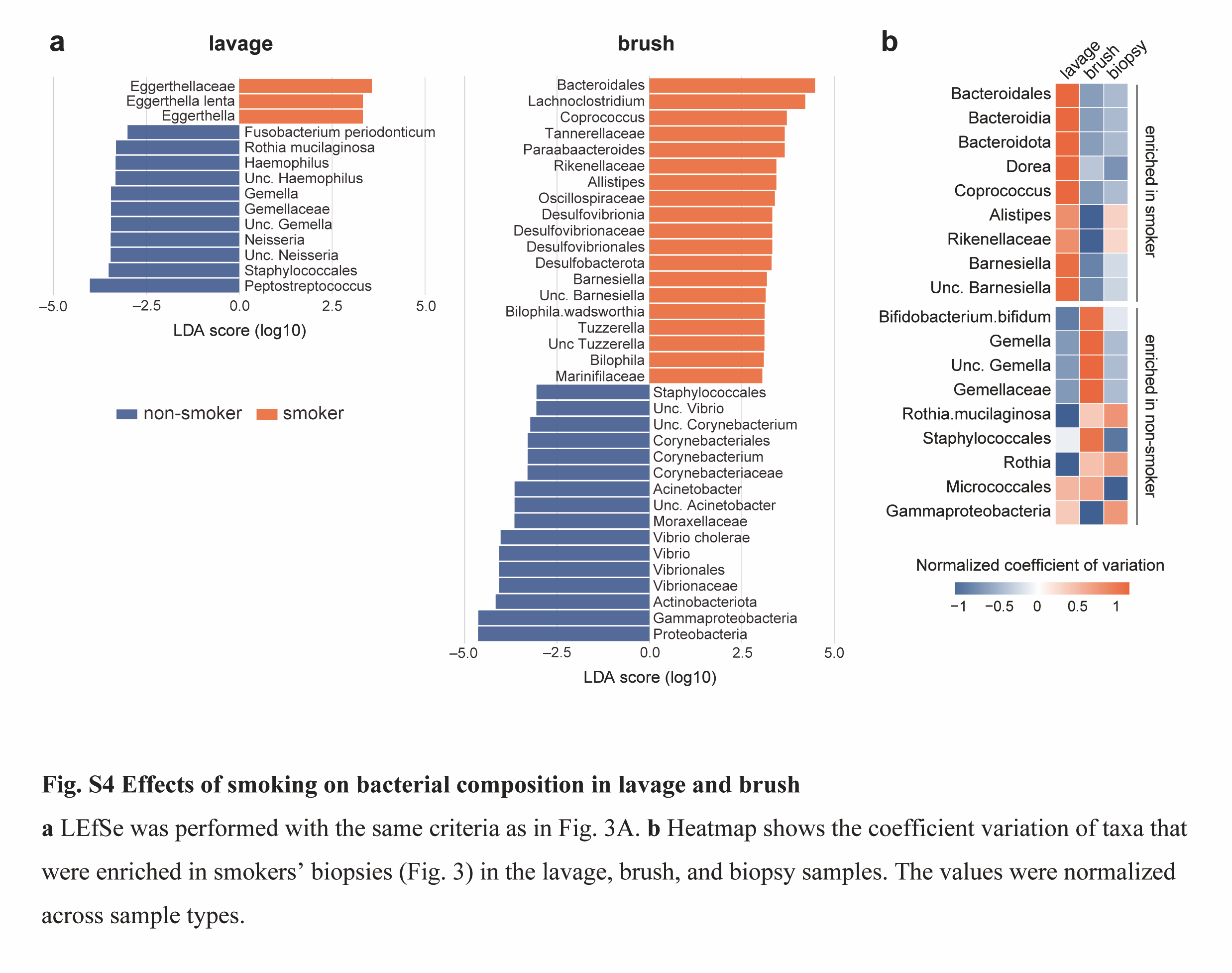

Supplement: Supplementary file 4 — Supplementary Information 4. [file 41598_2022_5936_MOESM4_ESM.tif]
